# Development and characterization of novel microsatellite markers by Next Generation Sequencing for the blue and red shrimp *Aristeus antennatus*

**DOI:** 10.7717/peerj.2200

**Published:** 2016-07-27

**Authors:** Sandra Heras, Laia Planella, Ilaria Caldarazzo, Manuel Vera, José-Luis García-Marín, Maria Ines Roldán

**Affiliations:** Biology Department, University of Girona, Girona, Spain

**Keywords:** *Aristeus antennatus*, Next-generation sequencing, Microsatellite loci, Population genetics, Genetic stocks, Blue and red shrimp

## Abstract

The blue and red shrimp, *Aristeus antennatus*, is a commercially important crustacean, in the Mediterranean Sea, which has been listed as a priority species for fishery management. Hypervariable microsatellite markers could be a useful tool to identify genetic stocks among geographically close fishing grounds. Potential microsatellite markers (97) identified from next-generation sequencing of an individual shrimp using a 454 GS Junior Pyrosequencer were tested on a preliminary panel of 15 individuals representing the four worldwide genetic stocks of the species from which 35 polymorphic loci were identified and used to characterize an additional 20 individuals from the Western Mediterranean Sea. In the Western Mediterranean sample, 32 out of 35 were polymorphic loci and the number of alleles per locus ranged from 2 to 14 and expected heterozygosity ranged from 0.050 to 0.968. No linkage disequilibrium was detected, indicating the independence of the loci. These novel microsatellites provide additional tools to address questions relating to genetic diversity, parentage studies and connectivity patterns of *A. antennatus* populations and help develop effective strategies to ensure long-term sustainability of this resource.

## Introduction

The demersal blue and red shrimp *Aristeus antennatus* (Risso, 1816) (Crustacea, Decapoda) is distributed in the Mediterranean Sea, eastern Atlantic waters adjacent to Gibraltar Strait and the Indian Ocean from Mozambique to Zanzibar (Fernández et al., 2013 and references therein) and has been recorded recently from the Brazilian coast (Serejo et al., 2007). The species inhabits the muddy bottoms of the continental slope along submarine canyons, ranging from 80 to 3,300 m depth, making this the most eurybathic species in the Mediterranean Sea, and has a complex demographic structure in the water column (Sardà et al., 2010 and references therein). Populations in shallower waters (<1, 000 m depth) which are exploited by commercial fishing are mainly composed of large and mature females with a low but significant proportion of males that return to deeper grounds after mating takes place, principally in the summer. Populations in deeper waters (>1, 000 m depth), which are not fished, have lower abundances and biomasses and are mainly composed of males and juveniles (Sardà et al., 2010).

Exploited since the 1950s, the global catch has increased annually, reaching 1,851 tonnes in 2013 (FAO, 2015). Currently the focus of an important trawl fishery because of its high value in Mediterranean fish markets (60–120 $/kg), neither international conservation strategies nor worldwide sustainable management policies have been developed for this species despite priority listing for action by the Scientific Advisory Committee (SAC) of the General Fisheries Commission for the Mediterranean Sea (FAO, 2006). An annual 60 days local closure in the fishing area of Palamós (Spanish Western Mediterranean) has been recently implemented, aimed at achieving the sustainable exploitation in this fishing ground (BOE, 2013).

Although studies have addressed *A. antennatus* biology and ecology, mainly of the Mediterranean populations (Fernández, 2012 and references therein), a major problem in achieving sustainable fisheries management is the identification of the biological units supporting the fishery, or the so-called genetic stocks (FAO, 1993). Mitochondrial DNA (mtDNA) diversity analyses of blue and red shrimp populations on a broad oceanographic scale have detected four genetic stocks: the Western Mediterranean Sea (WM), the Eastern Mediterranean Sea (EM), the Atlantic Ocean (AO) and the Indian Ocean (IO), but have failed to distinguish genetic divergence within the regional level (Maggio et al., 2009; Roldán et al., 2009; Fernández et al., 2011; Marra et al., 2015). Surveys using a small number of microsatellites isolated using the FIASCO protocol (Zane, Bargelloni & Patarnello, 2002) failed to distinguish any type of population structure within Italian fishing grounds (Cannas et al., 2008; Cannas et al., 2012).

Nevertheless, microsatellites have proved their utility in identifying genetic stocks in marine penaeoid shrimps around the world (Benzie, 2000; Brooker et al., 2000; Supungul, Sootanan & Klinbunga, 2000; Ball & Chapman, 2003; Maggioni, Rogers & Maclean, 2003; Waqairatu et al., 2012; Abdul-Aziz et al., 2015) and it is possible that a greater number of these might provide higher resolution in other Mediterranean and Mozambique populations. Next-generation sequencing (NGS) based on 454 pyrosequencing has enabled a high-throughput approach and has demonstrated its usefulness in the development of SSRs in non-model organisms (Schoebel et al., 2013; Sousa-Santos, Fonseca & Amorim, 2015). Long reads generated by 454 sequencing are expected to contain SSRs and their flanking regions that are needed to develop methods for SSR screening among populations.

The aim of our study was the development and characterization of novel polymorphic microsatellite loci for *A. antennatus* to provide additional markers necessary to analyze the structure and connectivity of blue and red shrimp populations at smaller geographical scales both geographically and bathymetrically, but particularly within the Western Mediterranean, and to provide insights into this species’ reproductive behaviour through enabling parentage analysis.

## Materials & Methods

### DNA extraction for next-generation sequencing

Genomic DNA isolation was performed from 10 mg of muscle tissue stored in 95% ethanol of an individual (Blanes 2010; specimen-voucher: LIGUDG:Aa:872, stored at our laboratory) by means of two series of Sambrook, Fritsch & Maniatis (1989) phenol–chloroform extraction protocol, with minor modifications. The sample was treated between the series with 1 µl RNase A (20 mg/ml; Invitrogen ™, Thermo Fisher Scientific) and incubated at 37°C for 30 min. The high quality of extracted DNA was checked by resolution on a 0.8% agarose gel with ethidium bromide (0.5 mg/ml) and was quantified using both a NanoDrop ND-1000 spectrophotometer (Thermo Fisher Scientific) (136 ng/µl) and Picogreen reagent^®^ (Thermo Fisher Scientific) (60 ng/µl) with a Paradigm Detection Platform (Beckman Coulter) (CEGEN-UPF Services).

### Next-generation sequencing and De Novo assembly

Two shotgun whole-genome sequencing was performed on a Roche 454 GS Junior Pyrosequencer by Pompeu Fabra University’s Genomics Service (Barcelona, Spain). The reads obtained were assembled into contigs using the GS De Novo Assembler program included in the Newbler software package v.2.7. (Roche 454 Life Sciences 2006–2012; Roche, Bramfort, CT, USA) using the default settings with two modifications to take into account that *A. antennatus* is a diploid (heterozygoteMode: true), eukaryotic organism (largeGenome: true).

### Microsatellite loci identification

To avoid redundancy with previous reported microsatellites, a BLASTn search employing our entire database and using a significance threshold of *e*-values <10^−10^ was performed against all published *A. antennatus* microsatellites (Cannas et al., 2008). All contigs matching with already available *A. antennatus* microsatellites were not included in the further microsatellite development. Remaining contigs were scanned with Tandem Repeats Finder v. 4.04 (Benson, 1999) to detect microsatellite motifs. Alignment parameters of match, mismatch and indels were set as 2, 7 and 7, respectively; minimum alignment score to report repeats was set to 30; maximum period size to 5 and matching and indel probabilities were set as 0.8 and 0.1, respectively. In addition, the option of flanking sequence was considered to record up to 500 nucleotides on each side of the repeat in order to later design specific PCR primers.

Contigs containing tandem repeats with at least 30 bp of flanking regions were used for primer design with Primer3 v. 0.4 (Untergrasser et al., 2012) using the following conditions: contiguous repeat areas that were less than 100 bp apart were considered the same locus (interrupted microsatellite), PCR product size ranged from 100 to 450 bp and PCR primers design followed the default general primer picking settings consisting of an optimal primer length of 20 bp (ranging between 18–27 bp), an optimal annealing temperature of 60°C (ranging between 57–63°C) and a GC content optimized between 20 and 80%.

### Verification of putative microsatellites

Putative microsatellites were preliminarily screened for amplification success and polymorphism by genotyping a panel of 15 *A. antennatus* individuals representative of four geographic areas showing significant genetic divergences with mitochondrial markers: WM (*n* = 5), EM (*n* = 3), AO (*n* = 4) and IO (*n* = 3) (Fernández et al., 2011). For each individual, the total DNA extraction was conducted following the standard phenol-chloroform procedure (Sambrook, Fritsch & Maniatis, 1989). The approach of Schuelke (2000), which included three primers in a nested PCR, was conducted for SSR genotyping. For each locus, we used a non-labelled specific forward primer with a universal 19 bp 5′-M13 tail (5′-CACGACGTTGTAAAACGAC-3′), a non-labelled specific reverse primer and the universal fluorescently 6-FAM-labelled M13 as the third primer. This reduced genotyping costs because only a common fluorescently labelled primer was required for all putative microsatellite verifications.

Putative microsatellites were amplified in 20 µl of individual (singleplex) PCR reactions containing 0.5 U of EcoTaq DNA polymerase (Ecogen, Barcelona, Spain), 2 µl 10× PCR buffer, 0.6 µl MgCl_2_ (50 mM), 2 µl dNTP (10 mM), 0.2 µM of both reverse and 6-FAM-labeled M13 primers, 0.04 µM forward primer and 1µl DNA template (30–50 ng) plus ddH_2_O. PCR reactions were performed on a MJ Research PTC-200 thermal cycler (Bio-Rad, USA) under the following conditions: an initial denaturation at 92°C for 2 min; followed by 35 cycles of denaturation at 94°C for 30 s, annealing at the optimal temperature at 50°C or 60°C (Table 1) for 90 s and extension at 72°C for 90 s; followed by a final extension at 60°C for 30 min.

**Table 1:** Characteristics of 35 new microsatellite markers developed for *A. antennatus*.

Locus name	Repeat motif	Primer sequence 5′–3′	Ta (°C)	*N*_*A*_	Allele size	GenBank
Aa1a	(AG)_7_	F: TTTCACGCATTTCTTGAACG	50	4	455–461(455)	KU195267
		R: GAGGAGCAGGGAGGTAGGTC				
Aa1b	(AACA)_7_	F: GTGTAGTGTCGTCGGGACCT	50	3	447–455(447)	KU195267
		R: CCGTAGATCGGCCTGTACTT				
Aa60	(TA)_57_	F: TTTGCCTTGCGATTTTTACC	60	3	197–313(307)	KU195268
		R: TTTCGGTTTCCAGAATTTCG				
Aa123	(AT)_10_	F: TACATGCGTTGGGAACGATA	60	6	444–454(448)	KU195269
		R: GGTGGTTTGCTCCATGATTC				
Aa125	(TA)_8_	F: GACTCGCTCGCACACATAAA	60	3	245–249(249)	KU195270
		R: AAATCTGGGTGTGCAAGGTC				
Aa127	(AC)_15_	F: TTTTGACGGTGGATCAGACA	60	4	351–373(371)	KU195271
		R: GTGTGTGCGCAACTCTCG				
Aa138	(AG)_23_	F. ACCAGCTCTCAGAAGGCTCA	50	11	217–249(231)	KU195272
		R: TGTTTCTGAGTGTGGCAACC				
Aa173	(TTTG)_5_	F: CCCAAAGCTCTAGCAACGTC	60	6	139–363(351)	KU195273
		R: GAGAGGAGATCGGTCTGTCG				
Aa243	(ATAC)_5_	F: CACCTTCAACAGCACACACC	60	3	352–360(356)	KU195274
		R: CCTTCATGACCTTGGCAGTT				
Aa268	(TTCAA)_5_	F: GCTTTTTGCAGGGCATGTAT	60	2	161–196(161)	KU195275
		R: TGAGTCAGCTGCCACGAC				
Aa274	(GTG)_31_	F: CTTCCTGGAGACAGCACACA	60	8	360–435(390)	KU195276
		R: ACGACACCTGCATCACCATA				
Aa315	(AGC)_5_	F: CTTCCTGTGCTCTCCTGGAC	60	2	417–423(423)	KU195277
		R: TCTCCGCATCTTCTTCGTTT				
Aa421	(AG)_13_	F: CAAACTGGATCTCTTCGCCA	60	7	197–251(205)	KU195278
		R: GGCAACTTTGTAATCTCTGTTGTC				
Aa496a	(AAT)_7_	F: AAAAGAAATAGGCCAGAACTGC	60	2	168–171(171)	KU195279
		R: GACGTTCCCTGGAGGTGTTA				
Aa496b	(AAC)_5_	F: TCCAGGGAACGTCCTTACAC	50	2	427–436(436)	KU195279
		R: GAGCCTGAGATCTTGGGTGA				
Aa510	(TCA)_5_	F: GTGGTAGCGAAACCTTTGGA	60	7	255–351(339)	KU195280
		R: ACAAGCTCGAGCCACTGAAT				
Aa590	(CTAAT)_13_	F: CCTCCCTGACACCCTGTTTA	50	5	393–523(408)	KU195281
		R: CCAGCATAGCCTTGATTTGG				
Aa667	(GAA)_5_	F: GCAACTGAGAGCCACAGTCA	50/60	3	269–275(272)	KU195282
		R: TTCAGCACGACTCTGGATTG				
Aa681	(CA)_21_	F: AATTGCGAAACTTCCGACAC	60	9	250–340(256)	KU195283
		R: AAAAGACGCAGGGAAGGTCT				
Aa691	(CT)_9_	F: GGTTGCCAGAGCACGATAAT	50	2	136–142(142)	KU195284
		R: ACATCCGCATTCTCTTCGAC				
Aa751	(AATTA)_5_	F: GTGTGAATCGACCGGAATCT	60	2	245–250(250)	KU195285
		R: GCGACAAAAGTGATGACCAA				
Aa785	(AAG)_6_	F: TATTTTTCCCTGGCTTGCAC	60	5	375–477(459)	KU195286
		R: ACCAGGCAGCCAGTCTATGT				
Aa818	(CAATT)_5_	F: GCGATCACTGAACAGGAATG	60	3	189–209(194)	KU195287
		R: TGGGTAAGTCCTAATGGACTGG				
Aa867	(AAC)_7_	F: GGCTTTCGATTGATGTTGGT	50	3	147–153(150)	KU195288
		R: TAGCCCCTGAGTGTGTGATG				
Aa956	(AGAT)_6_	F: CTTGCACATAACGCAGGTGT	50	3	219–231(231)	KU195289
		R: CGCATGTTGTGTTGATTTCG				
Aa1061	(AG)_7_	F: ACCGGCACTAGGTTGGACTA	60	4	208–224(212)	KU195290
		R: CGGACCATGAGCTTTCAAAT				
Aa1129	(CAT)_10_	F: TCAGGTGTGAGAGCAGGTTG	60	2	210–213(213)	KU195291
		R: TTAATGACCATGGCTTGTGC				
Aa1169	(CAG)_5_	F: TGTCACGCTTCCCAACACTA	60	3	243–261(261)	KU195292
		R: CTGCCTCCTGCAGCTGTATT				
Aa1195	(AGC)_33_	F: GGGGAGGAGCAGAGACAGTA	60	4	213–222(219)	KU195293
		R: ACGCTGTTACCTCGGCTTTA				
Aa1222	(TTCA)_6_	F: CGTTCGTCCTTTCTTTCCAC	60	3	160–172(168)	KU195294
		R: TTGTCTGCTCTTCCTATGTTGC				
Aa1255	(AT)_17_	F: TGTCACGTTTGGGTTTCTGA	50	5	144–158(158)	KU195295
		R: GCCGTGGTGGAATTTATCAT				
Aa1408	(ATTT)_5_	F: CCAGCCATACTCTGTCACCA	50/60	3	171–187(183)	KU195296
		R: TTTTGAGCGGGAAGTCATTC				
Aa1444	(AT)_10_	F: TTTGGAGAGTGGTTTGCTCA	60	7	197–211(199)	KU195297
		R: GCCATCACCACAACAACAG				
Aa1450	(AG)_7_	F: TCGGGAAGACGCTTCTTTTA	60	3	205–287(207)	KU195298
		R: AGAACACCCTGCCCTTACAT				
Aa1690	(TTGA)_5_	F: AGGACGACACTGTGTGTGGA	60	2	112–152(152)	KU195299
		R: TCTGCCAAGTTGAAACATCTG				

**Notes.**

Ta, annealing temperature; *N*_*A*_, number of alleles; allele size range (size of sequenced allele); GenBank accession number of the sequenced allele. Forward primers were tailed with M13 at 5′ and allele size range included 19 bp of M13.

Subsequently, 2 µl of amplified product was added to 10 µl of Hi-Di™ formamide with 0.1 µl of GeneScan™ 500 LIZ size standard and were loaded on to an ABI PRISM 3130 Genetic Analyzer (Applied Biosystems). Finally, allele scoring, after manually checking, was performed using Geneious v7.1.9 (Kearse et al., 2012).

### Data analysis

In order to assess the usefulness for population genetics studies, all loci yielding reliable polymorphism were further evaluated by genotyping 20 individuals collected from the same WM locality (Blanes) used in the preliminary screening. For each locus, observed heterozygosity (H_O_), expected heterozygosity (H_E_) and inbreeding coefficient (F_IS_) were estimated with Genepop v4.2.2 (Rousset, 2008) using allele identity. For loci where at least 70% of individuals were genotyped, departures from Hardy-Weinberg expectations (HWE) and linkage disequilibrium for each pair of loci were also tested using Genepop v4.2.2 (Rousset, 2008). Significance level was adjusted using Bonferroni correction for multiple testing. Micro-Checker (Van Oosterhout et al., 2004) was used to check for the presence of null alleles, stuttering or allele dropout in loci that presented significant departure from HWE. In addition, the combined non-exclusion probability test was conducted with Cervus v3.0.3 (Kalinowski, Taper & Marshall, 2007) to check for efficiency of these loci for inferring parentage analysis.

## Results and Discussion

### Next-generation sequencing and De Novo assembly

During the last decade, 454 pyrosequencing technology has been used widely to develop microsatellites in non-model organisms (Leese et al., 2012; Schoebel et al., 2013), as it is faster and more cost-effective than the previous methodologies (e.g., FIASCO protocol). The shotgun whole-genome sequencing with a Roche 454 pyrosequencer on the *A. antennatus* genome produced 165,507 reads with an average length of 343 bp (56,772,861 bp in total). Comparatively, the number of reads and total bp sequenced conforms to the values obtained by Leese et al. (2012) for other crustaceans, including decapods, using the same type of approach (49,802–186,890 and 12,098,817–55,152,741, respectively). Our average read length was clearly above the range of previous crustacean studies (211.5–265.5 bp). Up to 10,613 of the obtained reads were assembled into 2,029 contigs covering 895,246 bp of the *A. antennatus* genome and the number of singletons was 128,835. These contigs had an average length of 873 bp (range: 100–12,986 bp) and N50 contig size was 858 bp.

### Microsatellite loci identification and development

No homology with already available *A. antennatus* microsatellites was found in the BLASTn search, except for our contig00595 that matched the microsatellite Ant93 described in Cannas et al. (2008) (GenBank accession number EU417952).

In 247 contigs, a total of 317 tandem repeats were identified with Tandem Repeats Finder of which 241 presented motifs with a period size of di-(31%), tri-(27%), tetra-(24%) or penta-nucleotides (18%), repeated at least 5 times. The di-, tri- and tetra-nucleotides are the most common repeat motifs for microsatellite loci described in decapod shrimps (Scarbrough, Cowles & Carter, 2002), the di-nucleotides being the most abundant of them (Meehan et al., 2003; Robainas, Espinosa & García-Machado, 2003; Pan et al., 2004; Zeng et al., 2013; Baranski et al., 2014).

One hundred and ten of the putative microsatellite markers presented a minimum of 30 flanking bp, likely useful for primer design and Primer 3 software suggested specific primer pairs for 97 of these markers. These results were in the range observed in previous searches undertaken on crustaceans including decapods (25–1,420 putative markers) when stringent filters were used on massive 454 sequencing datasets (Leese et al., 2012). From the preliminary evaluation, 93 out of 97 microsatellites have amplified PCR products. After discarding primer pairs producing unexpected size fragments for microsatellite loci or which were monomorphic, 35 markers yielded reliable microsatellite variation with a total of 144 alleles ranging from 2 to 11 alleles per locus with an average of 4.1 (Table 1). Sequences of the contigs including these polymorphic markers were submitted to GenBank (Accession Numbers: KU195267 –KU195299) (Table 1 and Table S1). The proportion of success (36%) in detecting polymorphic loci for putative microsatellite markers was consistent to that achieved in decapods (15%–68%) (Meehan et al., 2003; Dao, Todd & Jerry, 2013; Dambach et al., 2013; Miller et al., 2013), in other marine species (e.g., 29% in a mollusk (Pardo et al., 2011) and also in fishes (∼40%; Sousa-Santos, Fonseca & Amorim, 2015; Williams et al., 2015)).

### Population genetic analysis

Of the 35 initial loci, 32 were polymorphic in the Western Mediterranean sample analysed with a mean number of alleles per locus of 4.8 (2–14) and a mean observed and expected heterozygosity of 0.350 (0.000–1.000) and 0.521 (0.050–0.968), respectively (Table 2). The number of alleles detected in our study resembled that previously shown in the Italian study (3–13; Cannas et al., 2008), but our estimates of observed and expected heterozygosity were broader than 0.2–0.85 and 0.23–0.89 in Cannas et al. (2008). In decapod shrimps, levels of polymorphism are usually high, with abundant alleles and often heterozygosity levels close to 1.0 (Scarbrough, Cowles & Carter, 2002).

**Table 2:** Results of genetic diversity of the 35 microsatellite loci in one locality from Western Mediterranean (*N* = 20) of *A. antennatus*.

Locus name	P	*N*_*A*_	Allele size	Ho	H_E_	F_IS_	*P*-value	*Null allele frequency*
Aa1a	95	4	455–463	0.684	0.643	−0.064	0.0947	
Aa1b	100	2	447–451	0.300	0.434	0.309	0.2804	
Aa60	5	1	307	0.000	0.000	–	–	
Aa123	100	5	444–452	0.700	0.645	−0.086	0.2435	
Aa125	50	3	245–249	0.200	0.589	0.660	–	
Aa127 ^a^	70	4	351–375	0.214	0.679	0.684	0.0016^*^	0.259
Aa138	100	14	213–249	0.750	0.921	0.186	0.0414	
Aa173	85	4	131–351	0.177	0.276	0.360	0.0694	
Aa243	100	5	352–376	0.500	0.458	−0.092	0.4902	
Aa268	10	1	161	0.000	0.000	–	–	
Aa274	65	12	363–468	0.231	0.968	0.762	–	
Aa315	100	2	417–423	1.000	0.500	−1.000	0.0000^*^	–
Aa421 ^a^	95	7	197–261	0.211	0.816	0.742	0.0000^*^	0.319
Aa496a	95	3	162–171	0.316	0.477	0.337	0.0194	
Aa496b	100	2	427–436	0.450	0.355	−0.267	0.5283	
Aa510	85	6	255–351	0.235	0.502	0.531	0.0070	
Aa590	85	3	398–523	0.471	0.443	−0.062	1.0000	
Aa667	100	5	260–275	0.650	0.732	0.112	0.1706	
Aa681	100	11	250–340	0.650	0.800	0.188	0.2607	
Aa691	95	3	140–144	0.263	0.240	−0.098	1.0000	
Aa751	100	2	245–250	0.200	0.390	0.487	0.0540	
Aa785	95	7	375–486	0.316	0.564	0.440	0.0117	
Aa818 ^a^	100	7	184–214	0.250	0.715	0.650	0.0000^*^	0.258
Aa867	100	2	150–153	0.000	0.100	1.000	0.0256	
Aa956	100	4	219–231	0.650	0.672	0.033	0.8058	
Aa1061 ^a^	100	5	208–226	0.250	0.703	0.644	0.0000^*^	0.253
Aa1129	100	2	210–213	0.050	0.050	0.000	1.0000	
Aa1169	55	2	249–261	0.000	0.182	1.000	–	
Aa1195	100	3	213–219	0.600	0.615	0.024	0.8929	
Aa1222	100	2	168–172	0.050	0.050	0.000	1.0000	
Aa1255 ^a^	90	6	144–158	0.222	0.672	0.669	0.0009^*^	0.255
Aa1408	100	3	171–187	0.200	0.190	−0.056	1.0000	
Aa1444 ^a^	95	9	197–221	0.3158	0.771	0.590	0.0000^*^	0.243
Aa1450	55	3	205–209	0.091	0.527	0.828	–	
Aa1690	100	1	152	0.000	0.000	–	–	

**Notes.**

*P*, Proportion of amplified individuals (%); *N*_*A*_, number of alleles; allele size range including 19 bp of M13; H_E_, expected heterozygosity; F_IS_, inbreeding coefficient; HWE, *P*-value.

* Significant departure from HWE for the loci with *P* ≥ 70% (see text).

a Locus with signs of null allele.

Among the 32 polymorphic loci, no linkage disequilibrium was detected after Bonferroni correction (*P* = 0.0001), suggesting their independent segregation. In 28 out of 32 polymorphic loci, proper amplification was obtained for more than 70% of the analysed specimens (*N* = 20). In 21 out of 28 the polymorphic loci, genotype proportions were consistent with HWE expectations after Bonferroni correction (*P* = 0.0018), with a mean number of alleles per locus of 4.4 and a mean expected heterozygosity of 0.455, which are in line with those obtained by Cannas et al. (2008) in 9 loci (5.3 and 0.637, respectively). Micro-Checker revealed null alleles (non-amplified alleles by PCR due mainly to mutations in flanking regions) as likely involved in significant heterozygote deficiency (positive F_IS_ values, see Table 2) observed in six loci. In addition, stuttering in two loci (Aa818 and Aa1444) contributed to genotype scoring errors. In most studies in other decapods, significant departures from HWE resulted in positive F_IS_ values (Supungul, Sootanan & Klinbunga, 2000; Ball & Chapman, 2003; Pan et al., 2004; Cannas et al., 2008; Dambach et al., 2013; Miller et al., 2013). Despite the majority of these authors arguing that the most probable reason for HWE departures was the presence of null alleles; this suggestion was only tested and confirmed by one study (Miller et al., 2013). In regard to Aa315locus in this study, all the analyzed individuals showed the same heterozygote genotype, and Micro-Checker failed to detect any genotyping error. It may be that, this is indicative of two duplicate regions; with no variation within regions but different fixed allele when compared (Moen et al., 2008).

The application of microsatellite markers also has proven its utility to study mating systems and paternity in decapod shrimps (Bilodeau, Felder & Neigel, 2005). In our study, using the 21 loci under HWE to test their potential in parentage analysis, the combined exclusion probability in parentage assignment was 0.9786 for a candidate parent when parents were unknown and the combined exclusion probability for the identity of two unrelated individuals was 0.9999. Thus, these results indicate the effectiveness of these loci for future parentage studies in natural populations of *A. antennatus*.

## Conclusions

Massive sequencing by a 454 pyrosequencing platform of the *A. antennatus* genome has allowed the development, validation and characterization of 35 new polymorphic microsatellite loci which greatly increases the number of available microsatellites from the eight microsatellites previously described (Cannas et al., 2008). This larger set of loci will provide a stronger set of tools to: (i) perform parentage studies, and (ii) examine connectivity patterns (horizontal and vertical) including examining the population structure of this species at a variety of geographical scales and, particularly, between fished populations in shallow waters and deeper unfished populations. This contribution will also assist to the responsible and sustainable management of this exploited marine resource, by means of a feasible strategy of temporal genetic monitoring.

##  Supplemental Information

10.7717/peerj.2200/supp-1 Table S1 35 polymorphic microsatellites of *Aristeus antennatus* Detailed sequences (contigs) contained the 35 polymorphic microsatellites of Aristeus antennatus.

## Additional Information and Declarations

### DNA Deposition

The following information was supplied regarding the deposition of DNA sequences: GenBank (accession numbers: KU195267–KU195299).

### Data Availability

The following information was supplied regarding data availability: The raw data has been supplied as Table S1.
