# Supplementary material for: Development and characterization of novel microsatellite markers by Next Generation Sequencing for the blue and red shrimp Aristeus antennatus
Source: PeerJ. 2016 Jul 27;4:e2200. doi: 10.7717/peerj.2200 (PMC4974933; doi:10.7717/peerj.2200)
Supplement: Table S1 — Detailed sequences (contigs) contained the 35 polymorphic microsatellites of Aristeus antennatus. [file peerj-04-2200-s001.pdf]

>*Aristeus antennatus* voucher LIGUDG Aa872; contig00001; microsatellite Aa1a and Aa1b sequences

CTCTCTCTCTCTCTCTCTCTCTCTCTCTCTCTCTCCCCCCCCAGTCGAAAGGGGGATGTGTC
CTCTTTGTTTGGTGTCGATGCAGGAGAGGGTCGCGAATGACTTCCCGCTCAAAACCTCTCTCT
CCCCCAGTCGAAAGGGAAGTGTGTCCTCTTTGTTTGGTGTCGATGCAGGAGGAGAGGGTGGC
GAATGACTTCCCGCCAAAAAAACTCTCTCTCTCCCCAGTCGAAAGGGGATGTGTCGGTGCAG
GAGAGGGTCGCGAATGACTTCCCGCCCAAAAACCTCTCTCTCTCCCCAGTCGAAAGGGGATG
TGTCCTTTCGTTTGGCGTCCATCCCTTCGCCTCATCCAGGTACGTGTCGCCGCTAAGTGTCCA
TTTTTCCACTTTGGGTCGCGTCCCCACAGAGAGAGGACAGGGGACGCCTCCTTCGGCCTGTC
TCTTTTTAAGGCAGGCGTCAAAAGTGACTTCCCGAATATGTTGTTGTTTTTTTAAGGGTACCC
AAGTGGGCCTCTCCCTTCCAGCCGACGGTAATATTCCCTCGTCCTCTGGGTTGGAAGGAGCT
CTCCCTCACCTATGCATTGGCGGACAGGGGAGAGTTTGAGAGGAACGGGGGCCAAAGTCAG
GAATAGGAGAAAAAGAAGCTATGCAATGTTTTTCGAGAGGAGGGACTCAAAATCATTTGTT
TCACACCCTGGGAAAGGCAGTAGTGGTGTCGTCCTAGCCCTTTATATGAACGAATTGGGAGG
GAAGGGAGTAGTCATGGCGTAAAACATGAATAATTCTCCTCCTCCTCCTCCTCTCTCCCTCCC
TCCGATTCATGAGCGTGTGTATTTTCACGCATTTCTTGAACGGGACGGGAGAGAGGAGGTGG
AATTCTTTTGGGGGATAGGAGAATAAGAAGCTGTTTGTTTTTTCTCTCTCGAGAGGACGGCC
TCAAAAAGCATGCGTTGAGATGTCATTCATTGGATCTGTACATACAGCCAACCCAGTTACTT
GGATAACGGTGGTGATTCCAATACATGCCTTCTTTTCTTTCTCATTTTCTAGAGACTCTGACA
GCAATATCCATGCCGTCCTAGCCCTTCAGGGCGTAAAGTAGTCACAGAAAagagagagagagagAA
AAAAAGAAAGGAGTCATGACGAAAAACTGTATCAGTCTCCTCCTCTCTCCCTGCCTCTAATA
ATTCATGCGTTTATTTTTTTCATTTTTATGCATTTCCTCGATGACCCAGCGCCACGCCACCTTA
TGACCTACCTCCCTGCTCCTCCCGAATCAGGATGAGAGAATAATGGCTGGTGGGTTGCTGGT
GTCAGGAAGGGAACCTTCCCCCTGGTGCCCGCCAGCGACTCTCTCGAGAGGGAGATACCGG
TGACGGGAGGGTCGGCGCGCAGTGGGAACGGGTTACAAGGGGAACCCCCCCTTCCTCAAGG
GTATCCATCCCTTCGCCTCATCAAGGTCCGTGTCGCCGCTAAGTGTCCATTTTTCCACTTTGG
GTCGCGTCCCCATCGGGAGTGGGCAGGGGACGTGTCCTTCGGCCTGCCTTTTTTTAAGGCAG
GTGTCAAAAGTGACTTCCCGAATATGTTGTTGTTTTTTTAAGGGTACCTTTTTGACCTCTCCCT
TCCAGCCGACGGCAATATTCCCTCGTCCTCTGGGTTGGAAGGAGCTCTCTCTCCCCTTTGCAT
TGGCGGACAGGGGAGAGCTTGAGAGGAGGGAGGCAAAATCAGTTCTTTCACACCCCCTCGA
AAGGCACTGACGTCCTAGCCTAGCCAGCAGCAAGGGATTCATGACGTAAAACTGAATCAAT
CGCCTCTTCTCTCCCTGCCTCCGTAGCATCAATTCATTTTGTTTGTATGGAATGGGAGAGAGG
TGTCGATTCAACAGGAGGATGTATATTTTCCGCTTGACCGTGCGGTACGTGCGTGTGTATTGT
ATGATATGCATTGCTGCTGTTGCATCCTCCGAAACCTGCTTGACATTTGCACGGGCCTCGCGC
CGGCCGGCCCGTCCTTTTCGTCTCGCAAACCCAACCCTTTCCATCTCTTGTTCTTTTCCCAGG
AGTATACGGTCACTCCGCCAGGTCCTAACCTGAACTATCACAGCACAAGCCCGCGGGCTCGC
ACCGGGCATGGATGAGTCACCCACAATAGCGGTGTTTCTCCTCTCCTCTAGCAGGGAGAGAA
GAATATACCCCTCTCTGCATCCATGCTCGGTCTTGCTCTTCAGATGCGGGATACCACTAGTTT
TCTTAGTTTGACGCCGGATGTATTCCGACCCAAACCCTGAACCGCGCGATCATCCCTTTGAG
ATCCTCCAGGCTCCTTTGCCCCACGAGCTAACTCTCGACAGGATGCGCGACGGGTCTCGGGC
AAAACTTAGATAAGGTCTGCGGACCTTCAGAGAAATAAAGAAAGACAGAGAATTGCAAAAA
GAAGAATTCTAGATGGCACTTTACATCCGCCCTACACCGTCTCGTGTACAGCACCAGGGGGA
GAATTCAGTAGGATTCTCCTCTCCTCTTAGGCATTTCGCCGTCAGAGAGCGCCAGGCGGGTC
CTCGTACCCCCCGGGCGTCCCTGCCGCTGCTGTACACACTGGACTGGGGGAAGGCCATGCAT
GGTGCCATGCATAAAATATATAAAAGGAAGGAAGGGCAGACGGAATACGCTCTCAGTTGGC
AAGTCATGATGATGATGATCCTCCTGTGTAGTGTCGTCGGGACCTCCTTCCAGAAGAATTCC
TACTTGGCATTTTCTGAATTTCTGAATTTCTGAATGAATGAAAGAAATAGTATTATGTCTTTA
GACATAAAACCAAGAAGGGAACGCGGAGTAGTACACACACACTCCTGCGTCCGTCCCAAGC
aacagacaaagcaaagaaacaaacaaacaAGCAGTCGTCACCAGAGAGATTGAGAGAGGAGAATATGATGA
TCCTCTTGTGTGCTGTTCTTGTTTTTCGTTTTTTGTTTTACAGGGAGGTAGGGGATCTTTCGGC
GGTGCTCGCCACACGCCGTCTGTCCCCATCCCCCCGCCAAAAGAAAAGAAAATTACCTACCT
GGTTGATCCTGCCAGTAGTCATATGCTTGTCTCAAAGATTAAGCCATGCATGTCTAAGTACA
GGCCGATCTACGGCGAAACCGCGGACGGCTCATTAAATCAGATATAATTCATTGGATCTGTA
CAACCCACTTACTTGGATAACTGTGGTAATTCTAGAGCTAATACATGCCTTTAGAGACTCTG
ACAGCAATGGAAGAGCGCTTTTATTAGTTCAAAACCCTTCGGACCTTGGGCCAAGAGATTTG
GTGAATCTAAATAACTTTTGCTGAGCGCACGTTCCTCGAATCGGCGCCGCATCTTGCAAGTG
TCTGCCTTATCAGCTCTCGATTGTAGGTTAAACGCCTACAATGGCTATAACGGGTAACGGGG
AATCAGGGTTCGATTCCGGAGAGGGAGCCTGAGAAACGGCTACCACATCTAAGGAAGGCAG
CAGGCGCGCAAATTACCCACTCCCGGCACGGGGAGGTAGTGACGAAAAATACTGTTGCGAG
ACTCCATCGAGGCTTCGCAATTGGAATGAGTACACTTTAAATCCTTGTACGAGGATCAAGTG

GAGGGCAAGTCTGGTGCCAGCCGCCGCGGTAATTCCAGCTCCATTAGCGTATATTAAAGTTG
TTGCGGTTAAAACGCTCGTAGTTGGCTCTCTGTTCTGGACTGGCGGTTCACCTTAGCGGTGCT
TACTGTACAGCTCCGAGCTATATCCCCGCCGGCTCGCCTGGGGTGCTCTTTACCGAGTGTCCC
TCGTGGCCGGCACGTTTACTTTGAAAAAATTAGAGTGCTCAGAGCAGGCTATACTAACAGCC
TGAATGGTGGTGCATGGAATAATGGAATAGGACCTCGGTTCTATTTTGTTGGTTTTCGGAAC
CCGAGGTAATGATTAAGAGAAGCAGACGGGGGCATTCGTACTGCGACGATAGAGGTGAAAT
TCTTAGACCGTCGCATGACGAGCTACTGCGAAAGCATTTGCCAAGGATGTTTTCATTAATCA
AGAACGAAAGTTAGAGGTTCGAAGGCGATCAGATACCGCCCTAGTTCTAACCATAAACGAT
GCTGACTAGCGATCCGCCGCAGTTATTCCCATGACCCGGCGGGGAGCTTCCGGGAAACCAA
AGTATTTGAGTTCCGGGGGAAGTATGGTTGCAAAGCTGAAACTTAAAGGAATTGACGGAAG
GGCACCACCAGGAGTGGAGCCTGCGGCTCAATTTGACTCAACACGGGGAATCTTACCAGGC
CCAGACACTGGAAGGATTGACAGATTGAAAGCTCTTTCTCGATTCGGTGGGTGGTGGTGCAT
GGCCGTTCTTAGTTGGTGGAGTGATTTGTCTGGTTAATTCCGATAACGAACGAGACTCTAGC
CTATTAACTAGTCGACGGATCTTACACTCCGTTGTTTTATATATATAAATGAAAATGGTGTCC
AGCTCGCAGCTTCTTCTTAGAGGGATAAGCGGCAGCAAAAAAAATACTAGCCGCACGAGAG
ATTGAGCCATAACAGGTCTGTGATGCCCTTAGATGTTCTGGGCCGCACGCGCGCTACAATGG
AGAGTTCAGCAAGCTCTCCCTGCTCCGACAGGAGTGGGTAACCTTATCAAAGCTGTCCTTAA
AGGGGATTGGGGCTTGCAAATGTGTCCCATGAACGAGGAATTCCCAGTAGGCGCAATTCAC
CAGATTGCACCGATTTAGTCCCTGCCCTTTGTACACACCGCCCGTCGCTACTACCGATTGAAT
GGTCTAGTGAGGGCATCGGACTGGCGCTCTTGGAGTGGTCTCTCACCTCGCTATGAAATCCT
CCTCGTGGGGACGAGAGGGGGGAGCCGCCTCGGGTCGACGGAAAGATGTCCGAGCTGGGTC
ATTTAGAGGAAGTAAAAGTCGTAACAAGGTTTCCGTAGGTGAACCTGCGGAAGGATCATTA
CAGTATTATATGTATTACACACAGAAATACATGATGATAAATAAAAGGGTCGCCAGGGGAC
CTGCACATCATCGTCGTCATCCTCCCCCTCCTCTGTAGGACGACGACGACGACGACGTCCGA
AGGCAGGTCCTGGCGCACACCCACCCCAATTATTGACATACCAATCATCAATCAATCGTGAC
TTCGATGACAGCTCCAAGGCAGGCAGATAGAGACAGTTATGTGTATTTGCAGACAGAAAAA
GCCATCCATCCATTCCCAAAAGGAGGAGGTGATGATGCTTATGCTGCTGCTGCTACTACTGT
GCTACTACTACTACTCCTTCTCTGTCGTCGTCGTCGTCTTTGATGATGATATCCTAGGAGGCT
TTAGAGGCTGCCTCCTTTGCCGCGCTGTTGCCTGGCGCGGCAAGAACAACAGACTGCTCTCT
CGCCTGCCTCTTGAGGCAGAACTCTCCTTTGAGGGTCTCCAGACTTCTTCTTCTCCTTTAATG
GTTGTCTAGAAGAAAGAAGGAACTCTTTATTCCATATCAAACCCTTTATTGGCTTCCCCGGA
CACAAGCGGGGATGATCATGATTGTGACTTCTGTCATAAACCCAAAGCACAACTCTTAACGG
GGGATCACTTGGCTCGTGGGTCGAGGAAGACCGTGGTAAAAAGCGAGGTTTGATGGCTATC
GCAATTTTCACGTGACTCCATCGACACGTCGAACGCACATGGCGGCGGCTGCCGTAAAGGCC
TAGTACACTACTAATATATAGGCTGGCGGTCGCCACCTTCACTCGAGAGTCGTTCCGCCCCTT
TGCACAGACAATGGGTGTGGATTATTCTTTTAAGAGTAAGATTATCGCCGTGACGCCGCACA
AGGCTCACCAAGGCCTCGTGCCTGCCGTTGCCCTGCAACGCCCTGGGGAGATCGCTGGCAAA
ATAAGTTCGGAGCGTGCCAAAGACAAATTCCCGCCAGCGTTCCCTTAAAACAAATCACATGC
ACGTGCACCTCCGACGACACAAGTTCCCGGCCGAGGGGGGAGCGCACAGCACTGGCACTCA
CTCAGAGAGAGAGACGGATAGAGTGAGTGCTTCTTGGGAGGCAACAGCCCCCAAAAAGAGC
CCTGTGAGGCTGCGACGACGACTGGGACGACGACTGATGACGACGACGAAAGGACTTGCTC
TCTTTTGCTGCCTGCCGTGGCGGGCAGCTCCCTTGTCCTTCTCCCTTGCGATCTCACTCTCACA
CTGCCCGCTGGAGTGGGAGACGCAGTGAAGGGGGGAATGGAAGAGGGAGGCGTTTTCGGCG
TTTGCAAATGGATGCATGCTTCTCTTTGTCATTTGCAGTAGGAGTCATCATCACTGATCCCAG
TCAGTCAAAGCCTTGGCTTGTGTGCGTCACCTCTCCTCGTCGGTTTCCTTGACCGAAATATGA
AGGAAGGAAGGCAGACGGACCACCGACTGTACTGTACAGTTACAGACAGACCTAGACAAAG
TGCTGGCACAGAGAGAGAGCACCAGACTGGTTTTGCTAGGAGCCTGGCTGGCTCGGGGGGC
CGGGAAGGAAGGAAGTAAGGAGGGAAATCTTGCTTACCCAGGCCATCCCCTCATGCCACGG
CAGTGTCTGTCAGTCAGTCAGTGAGTCAAAGTCTTGACTTATTCCTCAGTCCGAGAAACGAG
GGAGGAAGCGACATTATATGTCTTTCGCTCTCTGTCCCTCGACGACGTTAAACTAATTTTTTT
GTTAGTAACCCACCAACTATTTTTGAAGCGACCTCGAGTTGAGGGAGATCACCCGCCAAATT
TAAGCATATTAATAAGCGGAGGAAAAGAAACCAACAGGGATTCCCTTAGTAAGGGCGACTG
AAACGGGAGTAGCCCAGCGCAAAGCCTCGTGCTTCCTAACGGGGCACGAGGGGTGCTGCGT
TTGGGAGAGGTCTTGCACGCGGTCCCGCGCCGCCTAAGTTGTACATGAATGACTCTAATGCC
CAAGGAGGGTGTCAGGCCCGTGTGGCGGTAAAAGACCCCCGAAGGGGGGAAGGTACGGGA
CTGCGGAAAGGGCCTCTCCTTAGAGTCGGGTTGCCTGAAACCGCAACCTAAAGTAGGTGGTA
AACTCCATCTAAGGCTAAATACGACCACGAGACCGATAGCGAACAAGTACCGTGAGGGAAA
GTTGAAACAGAACCCTGTAGAGAGAGTTCAAGAGAACGTGAAACCGTTAAGAGGCTAAACG
GGTGGAGATTCGAAGATCGAACTGGGGGGATTCAGTTCGTTCGGTAGTCGGGAGGGGGTTTT
CGGGACCACAACAGCGACCCCCGAGCGTGCGGCTCTAACGTCGCAGTCCGCTCTAGGGGGC

GCGGCCCGTCCCCTGCCCGGCGCCGGCGGGCGATTTTCCCCCCGCAGTAGGTCGCCGCGATG
CGTTGGGAGGGCGTCTGAGGCCCGGGCTTTGGCAGGAACCGACGTCGTCGCTGTCGCTAAA
GGCTGCCCCCGTGGTAGTCTGACAGAAGCGGCGTCTGGGGATCCCCCGGTGTAACTCATCGT
TGCATTAGTAGCGATGATCCTTGTCTTGCTCTCCCGACGGTCGCTCCTTGGGGCGGCGTCGTC
TGTCTGTTCGCAGCAGCGGCGCTCGCCTTCGCGGTGTGCAAAGAGTCGAGGCTCTACCCGAC
CCGTCTTGAAACACGGACCAAGGAGTCCAACATGTATGCGAGTCATTGGGCTCACTAAACCC
ACAGGCGCAATGAAAGTGAAAAGTGCTTTGCCTCGTGCATAGCTACAGGGCCGATCCCTCG
GTCCGGACGACCTCTTGCTCTCCCTGGACGGAGTGACAAAACAATGCCTTCCTCCCCGGGGA
GCTACAGCTCTGTAGTACTCGGGCTAGGGGACTCGTCCGGTGCCGCAAGCCTCTCCTCCTGC
CTCCGGTAGGAGGAAGGGGGGCGCAGGTCCGGGACTCGCCATGACGAACAGCTGTCTCTCT
GGCTGGCTGCCTCAACAGTGGCTAGCTTGCGGAGCTTCTGGATGTGTCGGCGAGTACCTAGA
GCATAAATGTTGGTACCCGAAAGATAGTGAACTATGCCTGGCCAGGATGAAGCCAGGGGAA
ACCCTGGTGGAGGTCCGTAGCGATTCTGACGTGCAAATCGATCGTCAGAGCTGGGTATAGGG
GCGAAAGACCAATCGAACTATCTAGTAGCTGGTTCCCTCCGAAGTTTCCCTCAGGATAGCTG
GCACTCGTCAATAACGAGTTTCATCCGGTAAAGCGAATGATTAGGGGTGTTGGGGACGAAA
TGTCCTCAGCCTATTCTCAAACTTTAAATGGGTGAGGTGCCGGGCTTGCTTTATGTGCCCTCA
CGGGCCTTGAAGCCCCGGACACGAATCGGAGTGCTTAGTGGGCCAATTTTGGTAAGCAGAA
CTGGCGCTGTGGGATGAACCAAACGTCGAGTTAAGGGGCCTAAATGGATGCTAATTCCGAT
ACCATGGAAGGGAGTTGCTTGCTATAGACAGCAGGACGGTGGCCATGGAAGTTGGAATCCG
CTAAGGAGTGTGTAACAACTCACCTGCCGAAGCAAGTAACCCTGAAAATGGCATGGCGCTC
AAGCATCCTCCCGATACTCGACCGCCGGGCGCACTATTGGCTGCAACAAGGAGGTCTCGAG
GCCCCGGTGAGTAGGAGGAGCGCGGTGGTGAGCGTTGAAGGATTCGGCGTGAGCCGGTCTG
GAGCCGCCACTGGTGCGGATCTTGGTGGTAGTAGCAAATATTCGAGCGAGACCACCCTCGTT
GACCGACGCGGAGAAGGGTTCCACGCCAACATCGCTTGGTCGTGGGTTAGTCGTACCTAAGC
CCGGGGATAGCATCCCGATTTACTAGGTGTGCTGGCTTTCCTGTCCCAAGTCATAATTATGCA
CCACCCTGGTAATGAATGATATAGTCCTCCCTCGCGCCTCGCAAGAGTATTCCCCGCATCTCC
CTCTCTTTTTTTTCTCGAAAAGAAAGTAAGGGTGTTGAAGGAGAGTGCCTGCATTCTCCGTTG
TTACCTTGCGCTGGCGGAGGTGGACTGGCCCGCCCAGCCCAGGCGGAGCTTTGAAAGAGCTT
GATTTTATCCCTTGTCCAAATGTCATGCTCTAAGAAGACAGACAACAACTACTGTAATAGGC
AGACGGACAGACTTTCTTTCTTTCATTAGTCAGTTGGTCGGTCCGGTCGGTCATTCAGTTCGT
TCGTTGCTGTTGCTATCCATAGCATCATAGCGATACGCTGCTGCTGCTACTAGTTTTGAGAAG
AGAGAGAGAGATTGTCTCATAACACACAGCCAGCCAGCCGGCGATCGCTTGCTTGCTTCCCC
AAACGGTCTGTTTAAAGTCCCAGATCAGTGGCGAAAGGGAATACGGTCAATATTCCGTAACC
TGGCCCGGTACCACCCGCCTCCCTTCCAGCTGCTCATATCCCATGACCCCTTATCCCAACAGC
ATTCATTCATTCATTCGTGTCTGTTTGTTTGTAGTGTCCTCGAGGATAGGGGTCTAGAGAGTA
GCCTGGGAGGCGGGTTAAAACTTTTAAAACAATACAAGTTATTGTCGATGCCGAAGGCATG
GGAGTTAGTCACTTCCTTGCCGCATCTAAGGACCGGTCTGGCGACCTGTGTCGCTAGATGAT
TATCCTCTTCCAAAAGAAGAAACTGCTTTGATAGTACTTACTGCAGCTGCTTGGTAGCTACTA
CTACGACGACTGCTATTGCTGCTGCTGCAGCTGATACTGCTATTTCCAAAGCAGCTTGTGTGC
CCCCGTTCTCCTAAAGATGGCGTGTGTTTTTTTTGGGTGCCGTCCGAGACCGTGTGCCTGAAT
GTTTGAAGACACGGCTCGGCGGCAGGCTCCCCCATTTTGTGGTTGGCACTAGTTCGACTGGC
ACCCCGGTAACGGGAACCTGTTCGCTGACGCCGGCGGTGGTCCGGGAAAGAGTTTCTTCTC
CCTTTTAGGCAGCCGGAGACCCCTTAAAGTTTTCACTTGGCGAGGGGGTCGTTGGTAGTAAC
AATATGATTGGAGAGGCAATGCATAATGCCCCGGTGTTCGCCGTAGAGCGCCACGCAATTA
AGCGTAGGTGTCCCGTGCACTGCCGCCGGCCCTTGAAAAGGCGATGCGGGTTACCACTCGAG
CAAACCACTGCCACACCACTCAATGAAAAGAGCCCTTCAATGCCCTGACCATACCTCTGGGG
ACGGAGAGAGAGAAAGGCACACACACACATACTGGCTGGTAGTATTATTATTAGCTGTAGC
AAGCGAGAGAGAGAGCAGAGTAGTATGGTAGTAGCCGTAGTACTATGACAACAACGCAGTT
TCTGTCTCCTTCCTTTGGTTGATTGCTGTTGCTGGACGGACGCCCACAGCGTCTGTCTTCACA
CTCTTCTGAAGTGATCTGGCTTAGTCGGCCGACCTCCCATCCTCCGGGCTTGGGTTGGTCGTG
CCTTCTTGAGTCCTGTGTCCAAACTTTTAAAAGAAGCGGGACAGCCAGCCATCAGATGGAGT
GTGAGAGAGAGAGATAGCGCAGCCGCTCACCAGAGGAGGCAGAGGAGACAGGAGGAAAGG
ATCATAGAGGTCCCTTGGCATCGGGTCAGCACGTACCGAATCCGCAGCAGGTCTCCGAGGTG
TGGAGCCTCTGGTCGATAGATTAATGTAGGTAAGGGAAGTCGGCAAGTTAGATCCGTAACTT
CGGGAAAAGGATTGGCTCTGAGGGTCGGGTCCAGTCGGGCCTGCGCGGGAAGCGGCTCCTT
CGTACGTGGGCATCCTTGGTGCCTGTCTAAAGGAGAAAACGGACTGGGCGAGGGGCCGATC
GTCGCTTAGGCTTCCATTCCCTCCTCTGGCTTCATGCATCTTCTCGTGTTCCTGCCGCTGCTGC
GGCGGTCTCCGGTGCTCTTCTGGGGCCCGGGGGTCGTTGTTGTGGTGGTGTTGGGCCGGGGA
GCTCTTGTGTGTTCTAGGGGGTCCAGGGGTGGCGGTTCGAGGTGGCGTGGCGCACCGAGCTC
GGACCCAATCTGTTACGGCAGCATACCGGGGCCCTATCCATTAGGACCGTGGAATGCGTTTG

GCAAAGGGTGGGCTTTGTTGTCCCGTCGCTAACACCCTGTTGTGCGGCCGAAGGGGAATCAT
CATGGCCTTTGGTCGTCCCATAAGAGTTTTCATTAACTAAGCATTGCGGCCAATGGCATAAC
ATGTCCCCCGAGGCTCATGCACTGGGGTGGATGGGACGACCCCAACCGCGGCTGGCGGCTA
ACGGCCGTCTCAGAACTGGCACGGACCAGGGGAATCCGACTGTTTAATTAAAACAAAGCAA
TGCGATGGTCAGTGAGTGATGTTGACGCATTGTGATTTCTGCCCAGTGCTCTGAATGTCAAA
GTGAAGAGATTCAACCAAGCGCGGGTAAACGGCGGGAGTAACTATGACTCTCTTAAGGTAG
CCAAATGCCTCGTCATCTAATTAGTGACGCGCATGAATGGATTAACGAGATTCCCACTGTCC
CTATCTACTATCTAGCGAACCCACAGGCAGGGGAACGGGCCTGCAGTAAACAGCGGGGAAA
GAAGACCCTGTTGAGCTTGACTCTAGTTTGATTTTGTAGAGAGGCATCAGAGGTGTAGCATA
AGTGGGAGGCTTCGGCCGACAGTGAAATACCACTACTCTGATCGTTTCTCTACTTACTCGGTT
ATACGGGGACGGGAGCTTCCTCGCGAGGGGAATGCCTCCTGATTTTAGTTCAAAGCGGCAAT
GGTCGTTCGCGACCGCCGGCGACCCGTACCGAGGACAGAATCAGATGGGGAGTTTGACTGG
GGCGGTACATCTGTCAAATGATAACGCAGATGTCCCAAGGTCAGCTCAGCGTGGACAGAAA
CCACGTGTAGAGCCTAAGGGCAAATGCTGGCTTGATCCTGATTTTCAGTACGAATACGGACT
GCGAAAGCAAGGCCCAGCGATCCTTTTGACTTTTACGAGTTTTAAGCAAGAGGTGTCAGAAA
AGTTACCACAGGGATAACTGGCTTGTGGCGGCCAAGCGTTCATAGCGACGTCGCTTTTTGAT
CCTTCGATGTCGGCTCTTCCTATCATTGTGACGCAGTTTTCACCAAGCGTGGGATTGTTCACC
CTTTAATAGGGAACGTGAGCTGGGTTTAGACCGTCGTGAGACAGGTTAGTTTTACCCTACTG
TTTACTCGAAGCACGTTGCGACAGTAGCCCCGCCCAGTACGAGAGGAACGGTGGGTCCGGA
CCAATGGTCAACCTCTTGGCCGAGCGGCCAGTGGAGCGCAGCTACGTCCGGAGGGTTTACCC
CTGAAAGCCTCTCAAGGGTGAACCCATACTGAGCGTTAGCAACTTGACGTCGTGCGACCCAA
CGGATCGGCGAAAAAGTTTTTACGCGTGCCCCCTTTCCTTCGGGTCGGGGGGTGGCGGACTC
TGCCCATGCAAGGGGAACTCGGGGGCGGCATAAGAAAACGAGGCCGCTTTCCCTTCCCGAA
TACAGAGCCATCCAGATCCGGAGTGTCGTACCGAACCGTCTGCATACGACTTACGTATCGGC
CGGGGTGTCGTGATACCTAGAGCAGGGTAACTCCTGCGATTGGTTAAGACTCGTCCCCATTA
AGGTCGGAAGGTTTTGTCTGTCCCCAGAGACGGACACTAGGAGGTTACGCCCTTCCTCTTCT
TCTTAAGAATTAAGAAGAAGGGAAAAAACAAAAACAAAAAACATACAAGGAGAAGAAGGC
CCAGCGATCCTTTTGACTTAACAGAGATCTCTGAGAGACAGAGGAAAAGAAGTTGGAAGAA
AAACCCAAAAAACAAAAATTACCTGGTTTTATCATAATAACCTTCTTCTAATAAGAGAGAAT
GAATAAACAGGCGATGGGCATAATAAGCACCAACGTCCTCTTTTGGAGCCAGGGGAGCAGC
AGCAGCTGCCTCTTTTGCCCTCTGCCAGCTTCTAGTACTAAAAGTAAGTAGTACTGGTTGAGC
TTTCATTTTGTATAGAGAGAGGCATCAGAGG

>*Aristeus antennatus* voucher LIGUDG Aa872; contig00060; microsatellite Aa60 sequence

AAGTACGGGGTAGAAAAGAAACGTATGATGAGCACTAACAAGAATCGTCTGCAGTGATGGA
TCAGCAGGCTAGACCACGACGGTGGCGTTTGCCTTGCGATTTTTACCCTCCTAGAACCAATT
AACCATTTTCCATTGGTTTTAATAGTAATACTTTTGCGAAAATTAATTTCACAAGCTTTTTTA
GGAACCTATTACACGCGAAAGTACAGGTCCCACCGtatatatatatatacacagtatatagtatatatgtatatgtatatatat
tatatacagtatatacagtatgtatatatatatatgtataaatgtgtttaaatatgtatatatatataCAGTACATATCCTTTTATCCGAAAT
TCTGGAAACCGAAAAGCTCCGAAAACCGAACATTTTTGCACAAGAATATTTTGTTTATTTTTT
GGTGAACACATGTAAACAACTCCCACCCTGGCTCAGACTTTTCTCCTATTTTTCCCCAAAACT
GACCTTTAAAGGCAAGTTACATACAGTGTTGTTCATCTCAGCTGTTCAGCTCTTGTCTATAAG
CTCTCTAATAACGCCTATTGTCATGTGTTTATATCTTTAAATTGTTATATATTTAGTTCCAAAA
GCCCATTTAACGAGAAAAGTGGCCCACCCACTTGTTTAACGATATTTTATGGGGAAGCGAAA
ACTCTTCTCTCTGTCTCTACAAAAATAATAACAATCACCTAGACAAAGAGTAGAATAACTAC
AAGACCCACAGACCCATCGAAAGAAACTGAATAATTTTCACCTTCGACATCCAGATCGGCTT
GCGGAATACCTGAATAATAGTAGTTTTGTTTCATTGTCTCTTTCTCCCTCTCTCTCTTATGCGA
CTTTTTCAACAGTATACTAATGCTTCTTTAGACTGTAATTAAACCTTATTATGCATAAATAAA
CAATACAAAATGAATACGTATCTAAAAAATTACATTAACATGGAATGTGTCAGTGAGGGGG
GAAACTTTTTATGATGCAAACGGAATTGAGTCTTGGAGCGCTATGACATACGGTTCACTAAC
TTCACTTTGGAGAACATCAAGCCTCTTGTTGAAGGTTATTCGATAAGAAATTACTTCGATATA
GTTATAATAACGTTCAAAATATTTAAAAATCCTCTGCAAGAAGTATTATTGAATAGATGTAA
TGATATACACGAGAGTGAAAGTTTACGACGGGAAACAAACGCGCGCGCGTTACACCGATCG
CGAACGCTACGTGTCCAGCTGGCATGAAAGGGAATCATTCGTATGAACGTCTATTTCTTCAT
TTATAATGTTTATTTGATAAGAAATTACTTCTATATTGTTACAATAATGTTCAAAATACTGTT
CAGTACATCTGAAACCCCTCTGCTAGAAGTATTAGTGAAAAGAAGTAATAATATATATGAGA
GTG

>*Aristeus antennatus* voucher LIGUDG Aa872; contig00123; microsatellite Aa123 sequence

CAGGCACTTGCACTCTCTGTTCAGAACAGTGCCGTGTACATCTCTGAAGTGTTAGGACGTCT
GTCTCGTGAGCGACGCAGGGTATACTACGAACTTGTCTCTAACAACATTTCCATTGATGTCC
AGTAAGTGTTTGTGCAAAGTGATTTTGTGTGAGATTGGCGATAGATATATGTAAATTGCATT
TGTTTAAGGCGTAAATGTTTGTGTTTATGTACTGATATATACATGCGTTGGGAACGATAAGG
AGAAATGGATATAGAGATATAAATATGTTTATGTTGATGTATTTTAGGATTTCAAGGGATTT
GCGGCTTTATATATTAATATCAATATGTCGGAACTCTACCTAAGTCTTTCGAAAGACAACGT
AAATGTCTCTACCTATTTGGGAGGGCTAGATGTCCATGCTTTGCCTGAGGAAAAACACTGCA
GTCTTGTAAATGCGCTTTTAGTGAAAGTGTCTAAGAATCGAACTATTTGAAACTATATATTAT
ATACCATTGCGTAGGCatatatatatatatatatGAGTATCTATGTATATCTAGGAATATGTGTAGGTTT
TATATATATTAATTTTGTTTTATCTGTCTATCTTGATGTATGTTGGTTAGTTAGTTAGGTTAGT
CAGAATCATGGAGCAAACCACCACACAGGCCATGTGGGGCCCGTGAACTCTTTTTGATCTAT
GTATCTATATTATCTAGGTATCTAGTTATCTGCAGGTGGTAACAGCTTTGGCCAGAATAGGT
AAAAATAAGGCTTGTGGAGTTGATGAGATTCAAACGGGATTCCCAAGGCAAATAAATGATT
GTGAACGGAAGAAATGGTTTAACTAAAATGCTTAACTCGGTGAAGACAATTCACCTGATGAT
TCCAAAAAATATCAATATTTATGCTACTAGCCTAAAAAGTTTGGACAATCACTTTGAGAAGT
CATGAGTGTTCGATTTTGTTAACTGCAGTTATAGATATAATGTAAAGTATAGTAGAAAGCGA
GTTGTCGAATGTTCAGACCGGTTTTTAAAAAGGTAGAGGAAGGTAGGAGGGAGATTATTATT
GTTGTGCTCAAGAATTCTAATTAGAAGTGCAG

>*Aristeus antennatus* voucher LIGUDG Aa872; contig00125; microsatellite Aa125 sequence

CATGTATGACGTCATAAAACTTCCGGACTTCAATCACACTACCTGTTACTTAAGGTGACTCAT
TATTCCTTAAAAATAGCGAAAATCTTTGCAACGAGAAGGATCAACATGCCCCTCCACGACTC
GCTCGCACACATAAAGTACCATAAAGAACATCAATGTtatatatatatatataATGGTTTAATAATTAT
GATAAAGACCCACGAGCGCATACCGCCCATTTAGATGGGCGCAAATTTGTCAACTTTCATTG
TCCTCATTAAGCACAATTTGCCATCTAAAATTAGTTTTCTATAAATCATATTACAGAGAAAGC
GTTCAAGTTTGACCTTGCACACCCAGATTTTAATACCGAGTGTAAATACCGGCTGGTGGAGA
GGTGGAAAAAGCCACTCTGAGGAATTTCTCCAAAATGGCTCGCAATGATTACGTTTTATATA
ATTGTAGATATACACATGCATTCATTTCATGTAATAGAGGGTTTGTATAAGACACAAGGCCT
TACACTCATATATAAATGTGTTAGCTAAACTTTCATTCTTATATTATAGTAGCTGTTTGAATT
TGAAAATTGATGCCCATTCAAATGGGCATCATGGAGCCTGTGGGGAAAGGTGACCACCAGA
GATGAGTTTTCAGACAAGGCAGAATTAGCATTTAGTGATACTTTGTGGTACATACTCATGTTT
TATGATACCTTGAGTCGAGTTCTGCAAATTTAAGGGTTTAACACTAATCATGAAGTATATTG
GTTTAGATTTTTAAATTTAATTTTGTCTTTCTTGGTAGGTTTCTGAAGAATGTTGAGGAAGCA
GTTGACTATTTGTTTTCATTACCTGAAAAGGAAGGTGCCACAGTTAGCGACATATCACTATA
GCCTCCTGATGATGGGGCTGAGTCGGATGCTGATGACCCAAGTGAGGCTATTGTTGATGTTG
GGGAAGAAAATGTGAATCTACCGGGAGCCAGACTTTTAGAAAACCCTATGTATGTAGGTCT
ATTTTTTCCGTTTCTTCTTTGGTGGCTGAGTTCATCCTTCAGTTTGCTTAGGTGTAGTATGCTG
TGGCTCTTCAGGAATAATTACAGGCATAG

>*Aristeus antennatus* voucher LIGUDG Aa872; contig; microsatellite Aa127 sequence

ACACACACGTCCATCATCAGGAGTTATCTGAATCAGGAAAAAAGAAGTATAAACAGGACCG
AGTAATGAGTTTTCGATACTAAAACAGTTACATTTTAAAACGAACTAAAAAGGTCCGGATCA
AGTAAAACTATTATCGTACAAGAATGTAAAAAAGTTAAACAAATCATGCAAAAGTTAAATG
TAAAGGAGTATACAGATTAAGGAGACCGTCGGGTTTGGGGTATCGGGAGCGGTATATGGTC
TCGCATAATTGAGCATAAAAAGAAAACTATATGAGATATCGAGATGATTTTTTTGCATATGG
AAGAGCATGAAATTGCGCTTATTTTGACTTATGTTTTATTGAACAGAACTATACGGGGGCTT
AGCCAGCTCCCCCGAAAGTCAAAATGTTGAGATTTTGTGAAAATGAAAAGAAAGGTTTGCG
CTGTATGACCCCCCGTCATAACCGCTCATCAAAAATACATGGGACAAATATCATGAATAGTT
GAATCGTATACCTCGAATGTGTCGTAGGAGAATTTTTGAATTTTGCTTTACTTTTTGTGTAAA
TCAGTTTTAAAAAAGGGGCATTTTTTTGGGTAAAAAGTGAGATTTTGAAAAGGAATAACTCT
TATTTTTTCGAAATATAAAAATATCTCTACGACACATTTGAGCCATTTTATAGTACTTTCCAA
CAAAACAATCAGATTTCAAATCGGACAAGCCGTTTTGACGGTGGATCAGACAGAAGACGAA
AAAAAACGAAACCGAGAAAATGAAGTTTGTATTTGTGTCAATTTCATTGCTAATTTTTTTCAT
TTATATTGTATACTATATAATTGAAATGTATATATAAAAAAACGGGCAAATTATATACTTTTG
AATGCAATTTATTACAATACTCAATAAGCAATATTTTTCTTATTATGATTTTTTCGAAAAACA
CTTGGTTGGCAATGCGACTTTTTTCTTGATTTTCTTGAATAAAATAGCGCCCTATGCAAACAA
TGCTACTAAACAGCAACAGAAAacacacacacacatacatatacacacacGCGAGAGTTGCGCACACACA
CACACACACACACACA

>*Aristeus antennatus* voucher LIGUDG Aa872; contig00138; microsatellite Aa138 sequence

CCTACAAAACCAGCCTAAAACCATTGATTACTGCTCAAAAGAGAGTTATACGCACGATAGCT
GGATTGAAAAAGTACGACCACACGCATCAAAGCTTTAATAATCTAAAACTACTCAATCTTAA
TTATATCAACATCCATTGCTGTGTATTATTTGTCTACAAATCAATAAATCTATATGAAAATAG
TTTCTTTAGCTTTAGATATAATCAAAGGTATCAGCTGAGATACAGTAACCTGTTAAAACTGC
CATTTATAGGGTCCACTCAGGACGCAATCATGTATACTATACCATGGAGCAAAAATCTGGAA
CAATCTTCCTAACAGCGTTAGGAATAAATCAACAATAGGTTCTTTCAAACATGCACTTAAAG
AACATCTATGCTCCTCATTGAACTAATCAACCAAAAGCAATCGCAAAATTGCATTCGCCATC
TACTCTACTTCTCTATGTTTCATGTTACCTCATATTGTTGCATTATAAATATTACTAATTTGTG
TTCCGACTCTATCTAATGTTACCATATACTGGGTCAACTGCCACATGTTTCTGTACATTGATT
TTATTTTTCTATTACCACATATAATGTTAATTTACTATTTAACTGTCTTATCTTACCGCCATTT
GTTCTCGATCACTACATGTTAGTTCTGCCTCGACCTAAGCCTTATTTCCTTCTCTGTTTAATGT
TGCATTATCTGTTACATATTTTATTTTGTTTATTTAACCATAATTACATGTTCAATCTGTAAAC
ATAAGAATTAAGGTGCCTTCTCAAGAGAGCACCAGCTCTCAGAAGGCTCAGCCATATCATAA
TATGTACTGACTGTACTTGTATATAACAGCTTTGTAATACCTTTGGCTAAATAAACTTTATCA
ATATCAATATCAATATCAATATCAATACCACACCACACCACACCACACCagagagagagatagagagat
agaaagagagcgagagagagagagagaTCGAGGTTGCCACACTCAGAAACAAACGCTATAACGTCCTGGT
TCAACAGCGAGAGAATGCGGCCATGTCAGTGATATGGCGGCACCGACGTG

>*Aristeus antennatus* voucher LIGUDG Aa872; contig00173; microsatellite Aa173 sequence

GGGGACCTCGATGGAATTATATATGTGAGGGGCCCTAAGAGAATTTTCCATGGTCCTCTGGC
CTCCGATAAAAGGGTCCCCAATGGAATTATATATATATGAGGGGCCCTAAGCGAATTTGCGA
AGTGGCCCAACCGTAAGGGGGGCCCCCAAAGCTCTAGCAACGTCCCTGAGTAGAGTGGAAC
AAATTCTTCACAAACTTACACAACACACCTAAAAGAAGAGGATATTAAAAACTGTTCAAAC
CAAGATACAATGGTAATATTAGAGGAATTTATTTCAGCCAAAAAATAATTAAAAACAAGAA
TAAGATAAGATAAAAAACAAGTTAAATCATTGCCTGCGTTTAAAAACTCTTTAGACAGGTAC
TGGAAAAGCATtttgtttgtttgtttgtttgTTGGGGCTTTAGACCTATCAACTGCCATGGTCATTAAGGTC
AAAACACGGTCAACTCACGCACAACCGACAGACCGATCTCCTCTCACGGGAGATCAAAAGT
CCGTCTCAAACATAATTATGGTCTCACACCATAACTAGCCCATGAAAAAATAGCTGAGTGTT
CCACCGTCGGTATAAGGTCGGCCCAGAACGTACCGCAGGCGACCACGCATCAGAAGGATCA
CTTACCCGAGTCAGCCAAGCATCGCTTTTGGTTACTCCCAGTTACCACTCCGGAGCTAAAAT
CCATCATCCATGGTTCGAATTCATCATCTCCAGGCCACGACGACGTGTCATAGAGGGTCATC
AAGGACTGCGCACCGGAAATAGCCTCATTTCTTGAGTTTATCATTAACAGATGATTTAAAAC
CGGAACTTTCCCCGCCACCTTCAAGTCGCTCACGCGGCGCCGCCCCATAATAAGGGCGACGA
GTCCCGGCCTGACAACTACCGGCCATCATCAATCCTCCACAGTTTAAGCAGGATATTTGAGA
AGAGCGGTGGTGGGTAATAGGTTGA

>*Aristeus antennatus* voucher LIGUDG Aa872; contig00243; microsatellite Aa243 sequence

ACCAACACTATTCACCTACTATACCGCCCACCTACCACCTTCAACAGCACACACCAACTACA
TCTCATAATATCATATCTACACAACATCACACAAATCATCTCACACCCTTCCAAGTCAAAGC
ACATCATGAAGCTCGCCACTGAGAGAGCAATCTCACACATCAGAACATACGAACACGACTG
GAAGATACAGACAAACAGACATAAATTCAACATAATAAACGTGGCCCCACAGCCTCCCACC
TGCCCCACTCTACACATGGCTACGACGACCCCTGGCCCCCCGACCTGGACCACTCTAAatacata
catacatacatacTCTTTAGTTTGTTTGTTGGGACTTTACACCTACCAACTGCCAAGGTCATGAAGGT
CAACACTCACAACTCAAGAACAACCAAACAACCGATCTCCTCTCACGGGAGACCAAAAGTC
TATCTAAAAAGTATTTATGCTCTCACACCATAAATATGCCCATGTATATAGTGATATGTACGC
TATTTATTTATAGCTACACTGTACCTCCCACACCATTCAACCCATCTCTACCAAAACCTCCTC
ATCCCTTCCGCCCACCCCCTTAGCCGCACCTGACATCCAAAATCAAAATCAAACAAACAACC
AACGGCTTCCCGCCACCCCCACCCCACCGAATTATCTACGGTGACCCTTATCAAAGCCGTAA
ATCATGCCTCCACCCCCTCTTCCTCTTATCTCTCCTCTCACCTCTCACCTCCCACCCCCCGTAC
CAGCGACCTAAGGCGAACCTGAACATGTATATATCTATATTTTTTTTTCTTACATCTCTCCCT
ATGCATTTGTCTATATGTGTATATAAA

>*Aristeus antennatus* voucher LIGUDG Aa872; contig00268; microsatellite Aa268 sequence

TATTTCCTTTTGTGACAAGGTTGATAAGCTTGCCAAATCAGCTCTCCTACTAGAAGACCCTCA
TTATGTTGTACCGCTGTCTCTTATGCAGATGAAGCAACGCATTGTGAATTCCTGTAGAGATTA
TGAAGAACAGGCTTGGATTATAGAGAAATTAAAAGGCTCTGGCACCATCAGGCACTATGAG
GGTGTTGTTGGGACATCAGATAGATATAAACGCTTTAGGGTTTATAACGGTCTGTCCCGGAG
GCAACAGGTCACATTAACTAGGCTTCGCATTGGATATCAGTACACTACTTCCTATGTACAGG
ATGCAGTATTGGATGCCAAACCAAATTCATATCATTGTTGTAGACTCTGCAGATCACCTAGG
TCTCATGATCTCAATCATTATCTCCTTGAATGTTCCAAAACTGCATCTTATCGATCCAATAGT
GTTGTCCAAAGGACAACACTTATTGAACATGTCAAATTTATTATAGACACAGGTCTGATATA
TGATATAATTAATAATGTTAACGGTTTTCTACCGACCAGATAACTCAGACTAAGAAGGTGAG
CACCAAGGTGCAGCCCCTCAGGCATGTATGATCTAATGTTTGATTCATAGCCCTTTAATATAT
ATACGATCAGCCAGATTGGGTAGATGCCTCGGCATCTTTCCCTGGCTTTTTGCAGGGCATGT
ATTTGTTCTGTAAATAAATACTTttcaattcaattcaattcaattcaaCCGCTGAACGACGCGCCTCGCTGTCA
GCTCCCACCTTGCAGCTGAGGCGTGAGGGAGTCGTGGCAGCTGACTCACGTCC

>*Aristeus antennatus* voucher LIGUDG Aa872; contig00274; microsatellite Aa274 sequence

GGGGGGCAGAGGAGACGCACAGGATCGCTAGTGTTCTGAATATAGCTGAGGTACACTTTGC
CATTGGTATTAACAGTGCTGCCCTCCGAACCCAGGTGTGGATGGCGAGCGTTAAAGTCACCA
CACATGAGGACTGTACCACTACATATAAGACTATCGGGGGGCTTCGGTACCTCCCCGGTGTC
GTGGGGGTTGTAAACATTAATAATGGTGAGTGTTCTGCCCTCCAAGTACACATCTACACTGA
GGTACTCAACACGAGTTGTACCTCGCCCTTCCCTGGTCACTTTGAAGGGGATAGTGTTTCTAA
TATATATGCCAAGTCCTCGTATTGAAGGGTTAGTAGCATGAGGTTTGTCAACATATATATAG
CCAGGGATGTTGGCATACCGAGAACTAGATCGCACTTCCTGGAGACAGCACACATCTACACT
TTCCGAATGAAGATATTGGGAAAGTGTAAATTTACGTTTCTTTAAGCTGTTGATGTTCCAGGT
AACGCACTTGATGGAGTCAACGCCGAGCATCATTAGTTGAGGTGGTGAGGGATCCTTGATCT
GTGTGCTCCAGTTCCATGTTGGTAGTCTGATTGGCAGAAGGGTGTGGCAGCTGGTCTCTAGA
GACGGCGAGATGTAGCAGGAGCCGCTCCTGAAACTGTTGGTGTTgtggtggtggtgttgtggtggtggtggtgt
tgtggtggtggtgttgttgtggtggtggtgttgtggtggtgttgttgtggtggtggtgTTATGGTGATGCAGGTGTCGTGGTGATG
ATGCTGGTATTGTT

>*Aristeus antennatus* voucher LIGUDG Aa872; contig00315; microsatellite Aa315 sequence

CTTGTTCACGACAGAGTCATAACCTGTCATTGCAAAGACTAGCTATACACTCGTTGGTGATC
AACGAGAGGGCCGCCGAACCACCCTGGTCTGCCGGAACCTCGGCCTCTCTCTCGCACCAACG
AGTGCTTCCTGCGCCTTCAGCGCTGTCAGGCACTCGTGCCACAGCTGAAGCACCCACCACCA
CCGCCACACTTCGCCTACAAGGAATGCAGTAGCACGACAAAGATAGCAGCCACTGGCCCAC
TATCAAGTCGTGCTACAAGCGCCTTGTAGAGTATATTTCGAAGTCTTTTCCTCTGACTAAATT
CTTTCACTGCTTCCTGTGCTCTCCTGGACCTACCGAAGCCCTAGTGGCTTCAGTATTCGTCTG
GGGGAGGAATACCTCTCTGTTCCCCGTGTAGCCGTGGAACAAGGGGGGGTATCTCCAAGCA
CAAAGCCCCGATTCGGGAGGCAAAAGGGCGGCATCGCCCCACTGCCAATCGAGGGTCGGGC
TGTAGGCCAACAACTCCCTACACACACATAACATGTAagcagcagcagcagcACCAGTAACCGTCA
GCATGCCGAAGATTAAATTCGACGTGACTGGGGGCACCCCACCATCGGCAAACGACATGAG
CTACAAGCTGCACTATATCAAGCGAAAATCCGCCTGCTGAAGATCTTCCTGACACCAACAGC
ATTTATTGCAATCCTACAAAACGAAGAAGATGCGGAGAAATTGTTCACAAAGGAGGCCACC
ACCAAATTCCGC

>*Aristeus antennatus* voucher LIGUDG Aa872; contig00421; microsatellite Aa421 sequence

CTGATGCTAACATGCGATGATATCCATACAAACTGGATCTCTTCGCCAGTGCTGAATGCATG
TAGTATGTTACATTTGATATCAGCAACTAAGAGAGGTGACTCCTATTCGACTGAATTGGCAG
CCTATATAGCACTTTTGGAagagtgagagagagagagagagagagATTTGATTGATTTAAAACCTTTATTG
ACAACAGAGATTACAAAGTTGCCTAATCTTTGAAGTTTATATTACTAGCTAA

>*Aristeus antennatus* voucher LIGUDG Aa872; contig00496; microsatellite Aa496a and Aa496b sequences

TTGGCCACAACTCCCCCCTTCTTTAATAATAAAAGAAATAGGCCAGAACTGCCCCCCaataataat
aataataataatTATTATTCTTCTTCTGTCGCGAATACCTGTCTTAACACTTTTTTCAACATTTTTCTTT
CTTTCAAAAACAGAAACAGGTCAAAATAACACCTCCAGGGAACGTCCTTACACCTCAAAAA
AGAATCTGGTCACATTCCCACTCCAATCTGCATGCCTGCCTGCTCCTCTTCCTTCTTCCTTCTT

CTTTCTTCATAGCCGTCCGCCGTCCGCCGTCCGATGCTCCAATGCCCAGTTACGCACGGCACA
TCCTTCCTCTATCACCACCGCCGACGTCACAGTACAACACACCCACAACAACTCCACAAAAA
TACACCACCCCAATACTAAAACAGACCTACACCCCGTAATaacaacaacaacaacAAACTTAAAAAT
CACACAAAATTAAAACATCTCCTCAATACAAAAAAATACAAAAAAAACAAAAAACAATTAC
AACAACAAAGAGAAACACCATTATCAATAACAAACCTTACAAAAATGACACTCACCCAAGA
TCTCAGGCTCCCTCAAATAT

>*Aristeus antennatus* voucher LIGUDG Aa872; contig00510; microsatellite Aa510 sequence

ATGCGTATATCAGTAATGTAATTAAGTGAAGTATACTGTATATGTTATCATTAATATTATTAG
TTAATTAAATAATCAAATTGTTTTTTTACAAGGTGGTAGCGAAACCTTTGGAGGGTTAGGT
GGAGTAGGCTTCTGATTTAAAGGTAAAAACATTATACATATATAGAGGTGCCCTTACATTAG
GGCTtcatcatcatcatcaTAAACATTCGGCTCTCTTGTGTTGTGTGTATAGTGGTTCTGGTGGATCCC
CAAGTGCTCTGTGGAGGCTACGAGGCCACCAGTAGTGATCTCCAGTGATCTCCTTCTCCCTC
CTGAATGTTAGTGTAGCTGACATCCTCGGCGGCCTCCAGCCTCCCCCATGTTGTTTGTGCAGG
TGTGTGTATTCTAGTATTCAGTGGCTCGAGCTTGTATCGTGCGTGTTGTCCTCGTGCTGTGGC
TGTGTTGGGGTATTGTTGTCCGGAAATCCATTTTAGTGCTTTGTTTCGTATGGCTTGTAGTTTG
TGTTGGTTTGTTTTGGTGATTATGTGTAGTGGTGTTGGTGGGTATTCCATGATGAGACTGATT
AGTGTTTTACATAGCAGGA

>*Aristeus antennatus* voucher LIGUDG Aa872; contig00590; microsatellite Aa590 sequence

AGCGCATTAATTAATTACTCAGTTGATTCCTCCCTTACATCCTCACCCGGCACCGAGGGGCAT
CTCGGCACGAGGCTTGGAGTGGAAGAGATTCGTCAACTGATTCCCTCCCTGACACCCTGTTT
AACCTAACTGAACCTAATCTAACCAACCCCAATTTAACCGACCctaatctaatcaaacctaatctaatcgaaccta
atctaacctaacctaatctaacctaacctaatctaaCCGACTTATTGATCGAACAGATTTCTAAAGTATTAGTATAG
GCTACTGTACGATCTAGTCTCTCTATTACAAATTTGCACGGTATAACAGTTCCTACTTCCAGG
TACTAGAACATAGCCGATGGTTACTATATTAGTTTTTAATTGCAAGAATCACATCTCCGATC
ACTACTAAACTAACTTCTTCTTTTTTCTTTTTTTTTTTTTTTTAATAATGCCAGCAAGGCACGG
GCGAAGATCGCCCAAATCAAGGCTATGCTGGAGAAAAAAAAAAGGATAGAATCAGTCAAC
ACTTCTC

>*Aristeus antennatus* voucher LIGUDG Aa872; contig00667; microsatellite Aa667 sequence

GGTCTTCAAGCGACCGGGTTCGATCCTGCCCACGGGCCAAGGTTAAGAAGAGCAGACTCGG
GGTAAGGGTCTCCCAATGCCAAAACCAAAAAAGGGTGGGGGGGCGCCGTGGTATGGTGGTA
ACGTGCGCAACTGAGAGCCACAGTCAGTTGAGTAATGGAGACGGCTCATCTTCTATAAACA
GCGATCCTATATAGTAAATGGGAATAATAATGCTGCTGAGgaagaagaagaagaaGATTTAGGTGA
GGTTATTCGGCTTGGAAATATGAGAAATCTGAAGAAAAAAGTCACGGTTTTAATGTTTCTCT
CACGGTGGTAGCGACCAAACACGTGGGTGACTCAGCATATCGGGAACAATCAATCCAGAGT
CGTGCTGAACCACTCGAGGAGTGTGAAGAAAGAGCTGTTTGAGTGGTAGAATAAGGACATG
TTGACTGGACCATACGATGGGAATGATGAGATGGAGAGGGGGGAGGGGGGGTCTGGTATGG
AAT

>*Aristeus antennatus* voucher LIGUDG Aa872; contig00681; microsatellite Aa681 sequence

CGTTAACATCAAGTTTCTCGTCAAATTGAAGAAATCCGCGACGGAAACTTTTCAGTTATTGA
CTGAGGCTTACCTATAAAATTGCGAAACTTCCGACACGAACAGAGTCATATTACTTTTTACT
ATATGcacacacatacacatacacacacacacacatacacacgcacataCTCTGATGGCAGAGTGGGTTCACATCGGC
CAGATCGTAGATCAGCAGTATTATAGTGAAGTCTTGTCTGTAGAGTTTTTAGCCAACCAAAA
GGACACTGTGCTTGAGCACACACCTTATTCGCCAGACCTTCCCTGCGTCTTTTACCTCTTCCC
AAAGATCAAATCCATGCTCAAAGGAAACAGTTTTTTATCGGTAGAAGTTGTGAAAGCAAAC
ACGACGCAGCTTCTCATCAGCCTTACAGAACATGATAACAAAGTTACAGTCTGTCTCGTTTTT
TCTTTTGCTGTTGTATATAGGTGTGTGTGTGTGATCTTAGCTAAAAC

>*Aristeus antennatus* voucher LIGUDG Aa872; contig00691; microsatellite Aa691 sequence

GTAAAGGGCAAAAAAGAAATCATATATATCATATTATGATACGATTTCAATTATCTGTGTTA
CCATGGTTACCCTGTCAAAACAACCGCTATTACACAACTGATTTCCAACACGTCGCAGCCTG
CTTCCCTTATCAGGCACTAAGTCCAACATGTTCGATCATGAATCTCTCTACCCCTATGCTATA

TTTTTTGGATCTAAGGGTGTGATTATCGACAACTATTTTTTTCCAAATTTGAAATTTAAACCT
CCAAACTGATCATAAGTGATGTTATTTTGTCATTAGTGAGCATACAATTTGTAATTAGAATA
ATATAGACATTCCTTTCCTTTGAATCGGACGCAATGATGACGGGTAGGTTGCCAGAGCACGA
TAATCACACCCAGATCCGAAAAAATAATGTATAGctctctctctctctctCATCCTCTCACGAAGATA
TCCGCATACTATCTCCATCCGTCGAAGAGAATGCGGATGTCGAAA

>*Aristeus antennatus* voucher LIGUDG Aa872; contig00751; microsatellite Aa751 sequence

GATCCGCGACGGAATGGAGACGTGAGATAGTGTGAATCGACCGGAATCTTTTAGACTCCaatta
aattaaattaaattaaattaATTGATTCTATACAATGTTGCTTACTCAAAGTACATTTGAAAAAGGTCATTT
GTATTTACTAACAGAAAAAGGTCATTTGTATTTACTAACATTTCTGTGTTATCCTAATCAGAA
ATTAGATCTATATCATGAAAATAAGAAAATCAAAATAGTAATTGGTCATCACTTTTGTCGCG
TAGGTAAACTTTATCAAGCGCCAGTTGACACTTCCCTTACTTGAGAGAATGTGGTTATAATG
TTATAAACTTTTAATGCATAGCTAACGACTACTAAGATATGGTAGGTTAACAATTATGATCC
CTTCTTCCACTAGGTTAAGTGGACCGTCCCGCAAAATATGCAGGAAAATCACTATAAACGCA
ATAAAACAGATCCGGTCTTACA

>*Aristeus antennatus* voucher LIGUDG Aa872; contig00785; microsatellite Aa785 sequence

AGTTGCCTTATATTTTTCCCTGGCTTGCACAGTCATTTTAGTAAGATTAACCTTGGTTTTAATT
AAGATTGTAGAGGCTCCTCATACTTATTTGGAATATAGCTATATGGCAAGAGGAACATAATA
ATATAAATTACGTGAGAAATAACCAAGAAATAATGAGGATCTCGTCTCCTGAAACATTACCG
TGTTGTTATTTCAGCTGTTCAGCTGTTGTTTATAAACTTTGTAATAACATCTATTTTCATGTGT
TTATCTCTTTAACCCTTTCCATGAGTCTTTTTTTTCGATGGTGCTGGGAGGCGCAAGAAATAA
AAAaagaaaaagaagaagaagaaaTCATAGCATGCATTGCACAATGAAAGAAAACCAAGTTAAACTCT
CAGAGCTCGCCGAATAAGGAAGTCACAGGCCGCGAAACAATATTTACATAGACTGGCTGCC
TGGTGCCTATGTGTC

>*Aristeus antennatus* voucher LIGUDG Aa872; contig00818; microsatellite Aa818 sequence

GCAACCTCCGTACCCGACCTCACTCGCAGGTAACTAGCTCGTTACCACGTAACAACCATCCT
ACAACTCCTCCTAATGCCACCCTTGCGATCACTGAACAGGAATGATGAATGCACGCGTCCAG
TCCGAATAACACAcaattcaattcaattcaattcaattCTTTATTTATTAAAGTGTCACGATACATCTTTATGA
AAAATAAAATACAATATTTAAAACATCGAGTAAGGTTATGTACCCAGTCCATTAGGACTTAC
CCAGACACTATACATAGTGCAACATTTACATATCACCACATATACAGTATTATAATATAATT
AAAGCACCAAATAAAACAATTGCAATATAATATTAAAACAGAACAAAACGCATTTTACGTA
TGTTACAGTGAATATAGAACATAGTACGACATATATTGATAATAGGAAGAGGTATGATATAT
T

>*Aristeus antennatus* voucher LIGUDG Aa872; contig00867; microsatellite Aa867 sequence

TATTTTAACACAAGAGAGTAGGCATTCTGCTTTTACATGAAAATAAGAGCCAATGGTGTGGT
TTGTGAAAATAAACTTTAAAATAATCTGGGGGTAGGCCTAATGTTCTTGTAATAAGTTAGTT
CATCACACTGTCGTCACTGTCAGGGGATAAATAAACTGAGTTTATCTATTTTTTAAACAACC
ATTAAATTCCTATTTAAGGCTTTCGATTGATGTTGGTTGTGTGATGCTTTATGTTCTTGTCCTT
ATTGACCTGTCACTTTGGCAGCTGATAGGCCTCAaacaacaataacaacaacaacAGTTAGTTCATCACA
CACTCAGGGGCTAGATAAACTGCGAGTTTATCTATTTTTAGACTAATATTGAATTACTTTTTA
AGGATTTCAGTAAATGTCGGTTGTGTACGTTTGTG

>*Aristeus antennatus* voucher LIGUDG Aa872; contig00956; microsatellite Aa956 sequence

ACGCGTTTTCGTGCTCGTATGTGACAATATGTTCATGACTGTGTTATTGTTATCTAGCTATTC
CCCTCCCACACACTATCTAAAACACATATATTCATTCTCTCTCTCATCTATCATTATCTCTCTC
CTTAGTCACGTACGGAGAACACTTGCACATAACGCAGGTGTTACagatagataaatagatagatagatCTTT
ATTGACCACAAATTACAATGTTAAAATACACGTAAAGATATTAGATATATCATACATTCAAT
TTAAATGATGAGCTAGCTAGACATTTATGGTCCAAATATCACAATGCATGAATTATACAGAG
TTACCGAAATTATCACAACGAAATCAACACAACATGCGTTAAACCTTAAA

>*Aristeus antennatus* voucher LIGUDG Aa872; contig01061; microsatellite Aa1061 sequence

CCCCTGACGCCAACCCTCCCATTTACCCGGGCTCGGGACCGGCACTAGGTTGGACTAGATTG
TCCTCCCCAGTGGCTGGGTGAAagagagagagagagAAATTTGTGTCTGTGTCATCCATATTACCGG
AGTCGCACATTTTGTGGGTGCGCCCTGGAAGGTAATCCAGACACACATCTCATGTACACATA
ATTTATTGACTAACTTCAATAATTTGAAAGCTCATGGTCCGATGACCATTAAACACTTCCTGT
TTCTGTTTCTTATTCTGCCTTTATTTCCGCTCGGAATCATTGCTAAAACTATTTTCGTCAAGCT
AAGGGCGCTCTCAT

>*Aristeus antennatus* voucher LIGUDG Aa872; contig01129; microsatellite Aa1129 sequence

GGGGTATGTTGTATGTCCCAAGTATCAACTCGACTTATGCTACTGTAGAGTCGTGGCCTCAG
GTGCACCAAGGCTGCCCGTGAACACACTTGTCAGGTGTGAGAGCAGGTTGTCAACCTTGTCT
TCCGGTCCTGTGGCTGTGGTGGTTGTTGGAGTGTTCGTATCTCTTGGCTGCTCcatcatcaatctcatcat
catccaatcatcatCACAATCGGTGATTTAGTTTAGTTTGTTTGTTGGATTTATGGCTATCAACCGTTG
GCACAAGCCATGGTCATTAAGCCAAATCCTATTTATTCTGACTATCACAT

>*Aristeus antennatus* voucher LIGUDG Aa872; contig01169; microsatellite Aa1169 sequence

AATGTCACGCTTCCCAACACTAACGCTTATATAAACTTCTCCCTTATTACGCTTTTCTATGAT
TGGACCCCCAGCAGCTCTAACACCAACACATCCTACAGCTCCAGCTCCACCAGCGACTTCTTc
agcagcagcagcagCGACGACACCCGAGTTGACCACCCCAGGCTGTACATTGTACTTGTACTTTGTA
CACACTGGGGCAACTGCGGGTGTACACTGTACTTAATACAGCTGCAGGAGGCAGAGCTGAT
CCAAAGGCCTAAAATAGGCCAGGACGGAGCACCTTCAGGTGCATACGC

>*Aristeus antennatus* voucher LIGUDG Aa872; contig01195; microsatellite Aa1195 sequence

CACCGGACATGTCGCGGGCACAGCCTTCAGGGACACCGGGTGGAGGGGCAGGGCTGGGGAG
GGGGAGGAGCAGAGACAGTAGAAGCCAGAACCGGGATGGTTAGATACGGATGagcagcagcagca
gcagaagcatcggaagcagcagcagcagcagtagtagtagtagtagcagcagcagcaacagcaggcagcaccagcaccagcagcagcGTGG
GTATTGATCTAAGGTTTGGTCCTTAAAGCCGAGGTAACAGCGTGAATAGCTTGGTGGTGGTG
CGGGGGGGGAGGAGAG

>*Aristeus antennatus* voucher LIGUDG Aa872; contig01222; microsatellite Aa1222 sequence

TTAATTATTTTATATTACGTTATTCGCTTATCTTCCTGCACTAGTTTTTGTTACGTCATACTGA
TGCTACTCCGCTTGTTATTCGCTATTTCACTTTGCGACTTTTGTTCGTTCGTCCTTTCTTTCCAC
CATTTTCTTTCTATTTACCCTCTCttcattcatcattcattctttcaCTTTCGACCAAGACTGTGTAACCTCACT
TTACATCAATATAGCAACTGCCTATAACACTAGCAACATAGGAAGAGCAGACAAATAGTTA
ATGTCATATATATAATGAATG

>*Aristeus antennatus* voucher LIGUDG Aa872; contig01255; microsatellite Aa1255 sequence

TAATTAGGCAGCTGGTGTCCTCAGTCTGCTAAGTGTCACGTTTGGGTTTCTGATACatatatatatata
tatatatatacagtatatatatGCTCTGGGGGGTGCGGGGAGGGAGTCATGGATGACGGGTGGGGTGGGCG
GTGTATCATGATAAATTCCACCACGGCCGAACCAAGATGAGTTAAGAATTTAAAATCTATTT
CTGAATCCAGTTTGATGTGCTCTTTCATTTTGCAAGATATAAATTGGATTGGATCATTTTGAA
AATTCTG

>*Aristeus antennatus* voucher LIGUDG Aa872; contig01408; microsatellite Aa1408 sequence

CCAGCCATACTCTGTCACCATCGACCTCCGTCTCGCCGCTAAAGTGTCCATTTATTTATTTAT
TTTTTACTTTGTGTCACGGCCCCAGGGAGAGTCGAAAGGGGATGTGACCTTTCGTTTGGTCTC
GATGCAGGAGAGGGTCGCGAATGACTTCCCGCTCAAAACCTGTCGGtctctctctctctctcACACACA
CACACACACACACACACACACACACACACACACAC

>*Aristeus antennatus* voucher LIGUDG Aa872; contig01444; microsatellite Aa1444 sequence

AATTTGGAGAGTGGTTTGCTCACTTACCTAACTCCACAAACTCTTACTTTTTAGCCTAAATGA
GTGAGCTTTGGCTAATGTAGCACAACatattatatatatatatatGAATGATTATATATATCTCTTTCCCTC
TCTCTTTCTCACTCTCAACAGTTGTTGCTGTTGTTGTTGGTGATGGCTGTGGTGGTGGTAGTG
GTGGCGTCTGTAGTGGTAGTGGTGG

>*Aristeus antennatus* voucher LIGUDG Aa872; contig01450; microsatellite Aa1450 sequence

AGCAGTCGGGAAGACGCTTCTTTTAATCTTTTAGGAACTTCGTAGTGCCTTCCATCATCAGTG
AGTGATTAGTTGATTAAAGTTGGCCATTGGAAAAAACGATAATTGAATTCCagagagagagagagA
TTTAGTGAAAACCTTATTGACAGTGCAGTTATTACATTAATATGTAAGGGCAGGGTGTTCTA
CATCTGATCCAATAAAGGGTTCTTGGTAGG

>*Aristeus antennatus* voucher LIGUDG Aa872; contig01690; microsatellite Aa1690 sequence

GTACAGAACTATCTACAGTTCGTAAATATAGGACGACACTGTGTGTGGATGAAGGTGAAGA
CAGTGTGCGTATGAGTGATGGTttgattgattaattgattgaATTTTATAGCCAGAAGTGTACATAAATAC
TAAAGAATACAGATGTTTCAACTTGGCAGATTAG
